# Empowering primary care workers to improve health services: results from Mozambique's leadership and management development program

**DOI:** 10.1186/1478-4491-6-14

**Published:** 2008-07-23

**Authors:** Cary Perry

**Affiliations:** 1 Management Sciences for Health, Cambridge, MA, USA

## Abstract

This article is the third article in the Human Resources for Health journal's feature on the theme of leadership and management in public health. The series of six articles has been contributed by Management Sciences for Health (MSH) and will be published article-by-article over the next few weeks.

The third article presents a successful application in Mozambique of a leadership development program created by Management Sciences for Health (MSH). Through this program, managers from 40 countries have learned to work in teams to identify their priority challenges and act to implement effective responses.

From 2003 to 2004, 11 health units in Nampula Province, participated in a leadership and management development program called the Challenges Program. This was following an assessment which found that the quality of health services was poor, and senior officials determined that the underlying cause was the lack of human resource capacity in leadership and management in a rapidly decentralizing health care system.

The program was funded by the US Agency for International Development (USAID) and implemented in partnership between the Mozambican Ministry of Health (MOH) Provincial Directorate in Nampula and Management Sciences for Health (MSH). The Challenges Program used simple management and leadership tools to assist the health units and their communities to address health service challenges.

An evaluation of the program in 2005 showed that 10 of 11 health centers improved health services over the year of the program.

The Challenges Program used several strategies that contributed to successful outcomes. It integrated leadership strengthening into the day-to-day challenges that staff were facing in the health units. The second success factor in the Challenges Program was the creation of participatory teams. After the program, people no longer waited passively to be trained but instead proactively requested training in needed areas. MOH workers in Nampula reported that the program's approach to improving management and leadership capacity at all levels promoted the efficient use of resources and empowered staff to make a difference.

## Introduction

Health units in the Nampula Province of northern Mozambique are located in remote areas far from the Provincial Directorate. Directives and funding from the central MOH in Mozambique's capital, Maputo, arrive slowly and sometimes not at all. Mozambican health workers operate in areas of striking poverty, and managers work diligently to stretch out their resources in an environment with below-average health indicators, even for sub-Saharan Africa. Infant mortality is high, and the HIV infection rate had by 2002 climbed to a sobering national average of more than 13%.

In 2002, senior administrators in the MOH identified a lack of leadership and management capacity at all levels as a cause of the low quality of health service delivery. They recognized that to improve health services and the health of the Mozambican population, especially in a newly decentralized health system, they would need to invest in human resources. Problems included a lack of communication between the provincial and district levels; low employee morale; high staff turnover; a large demand for services combined with a constant shortage of personnel; and planning that was limited to lists of activities rather than coordinated processes with measurable outcomes and procedures for monitoring performance. The challenge at the health unit level, therefore, was to empower employees to achieve change using simple tools in a very low-resource environment.

### The Program

From 2003 to 2004, 11 health units- representing six health districts – in Nampula Province participated in a leadership and management development program that the health unit staff called the Challenges Program. The program was implemented in partnership between the MOH Provincial Directorate in Nampula and MSH. The Challenges Program used simple management and leadership tools to assist the health units and their communities to address health service challenges in family planning, maternal and child health, and basic hygiene and biosecurity.

The Challenges Program in the health units was launched during a two-day Leadership Dialogue in Nampula Province for 25 provincial and district health managers. The goal of the Leadership Dialogue was to develop a sense of ownership of the program by provincial, district, and health unit managers. The dialogue also allowed the program implementers to gauge the appropriate level of the workshop materials for district and health unit participants, and it succeeded in communicating to participants and facilitators that this program was about working in teams to achieve results.

The program comprised four workshops of three to five days each, with a fifth for evaluation. Each health unit sent two or three staff members to the workshops. The first workshop consisted of a combination of lectures, discussions, individual and group exercises, and self-assessments of leadership competencies. The topics included:

▪ Concepts of leadership and the MSH Leading and Managing Framework

▪ Leadership strategies

▪ Organizational change

▪ Communication, coaching, and mentoring

▪ Negotiation

▪ Human motivation

▪ Teamwork

▪ Action planning.

Afternoon sessions focused on applying the theoretical material to addressing health challenges. Participants learned to use management and leadership tools such as the Leading and Managing Framework, gap analysis, a priority matrix, the SMART Objectives Tool, an action planning template, and a client survey.

## Discussion

MSH staff and provincial facilitators visited each health unit at least monthly after the first workshop to support the participants and assess progress. The MSH philosophy of developing leaders at all levels meant that everyone from medical technicians and nurses to drivers and housekeeping staff participated in the program. Despite low literacy and staff inexperience with being included in decisions, all but one of the health units successfully built teams, applied the tools they had learned for selecting challenges and developing action plans, and reported on progress at the end of the year. In 2005 an evaluation revealed that the five most successful health units achieved the following in one year:

▪ one unit increased the percentage of attended births from 25% to 35%;

▪ one unit increased the achievement of basic hygiene and biosecurity standards by 67%;

▪ another unit reduced waiting time for pediatric visits by 2.5 hours and reduced errors in inpatient registries from 8.6 to zero;

▪ one unit constructed a biomedical waste container;

▪ another unit built a kitchen.

The Medical Director of Nampula Provincial Directorate noted that these "simple projects ... made a huge difference in the quality of care... Health care improved greatly for clients in the health units – the image of the health units improved. What also improved was the confidence of the population in the work of the health units."

## Conclusion

The Challenges Program used several strategies that contributed to successful outcomes. It integrated leadership strengthening into the day-to-day challenges that workers faced without taking them away for long periods of expensive training. The program also offered decentralized health units the opportunity to work with communities to address the communities' needs. Despite having no operating budget for much of the program, 10 of the 11 participating health units were able to achieve most of their goals. In the process, the program created a culture of results and gave managers and health care providers a sense of control over their actions.

The second success factor in the Challenges Program was the improvement in employee morale through the creation of participatory teams in a traditionally hierarchical structure. After their participation in the program, people no longer waited to be trained but instead asked for the training they needed. Overall the health units that participated in the project have taken a more active and positive role than before to overcome their challenges. The director of Mossuril Health Center commented that "What I noticed from the very beginning was that the staff was very motivated and they are still motivated because ... when they are called to participate ... they really feel considered, valued."

The most critical limiting factor for the health units was the lack of resources. Chronic delays in the disbursement of funds affected how ambitious the action plans could be. The general low level of services forced staff to focus on basic challenges such as improving cleanliness, improving biosecurity, decreasing patients' waiting time, and increasing the number of attended births. The health units also had few skills in developing indicators to monitor performance, although by the end of the program they proudly presented simple graphs and tacked them up in the health units for patients and staff to see.

MOH workers in Nampula are realistic when they speak about Mozambique's substantial health challenges. But they also report that the Challenges Program approach to improving management and leadership at all levels has promoted the efficient use of critical resources and, most important, empowered staff to make a difference in their own areas.

## Competing interests

The author declares that they have no competing interests.

